# The collaborative working group method for pre-trial knowledge mobilisation: a qualitative evaluation of a structured process for iteratively refining a complex intervention (DAFNE*plus*)

**DOI:** 10.1186/s40814-024-01576-3

**Published:** 2024-12-21

**Authors:** J. P. Breckenridge, R. Gossage-Worrall, P. Chadwick, N. De Zoysa, J. Elliott, C. Gianfrancesco, K. Hamilton, S. Heller, J. Lawton, D. Rankin, S. Stanton-Fay, E. Coates, On behalf of the DAFNEplus Collaborative Working Group, Susan Beveridge, Elsie Friel, Helen Rogers, Stephanie Amiel, Emma Smith, Debbie Cooke, Anita Beckwith, Liesl Richardson, David Hopkins, Alison Cox, Carolin Ferguson

**Affiliations:** 1 School of Health Sciences, University of Dundee, Dundee, UK; 2 Sheffield Centre for Health and Related Research, University of Sheffield, Sheffield, UK; 3 UCL Centre for Behaviour Change, London, UK; 4 Department of Oncology and Metabolism, The University of Sheffield, Sheffield, UK; 5 Centre for Population Health Sciences, University of Edinburgh, Edinburgh, UK; 6 Kings College Hospital, NHS Foundation Trust, London, UK; 7 Sheffield Teaching Hospitals NHS Trust, Sheffield, UK

**Keywords:** Co-design, Collaborative working, Feasibility study, Intervention development, Knowledge mobilisation, Pilot study, Process evaluation, Qualitative research, Type 1 diabetes

## Abstract

**Background:**

There is a lack of practical guidance about how to effectively mobilise knowledge at the pre-trial stage. Despite increased guidance on developing complex interventions in recent years, much of this focuses on the theory and principles behind high-quality intervention development, rather than the practical aspects of how this should be achieved. This paper shares the findings from an embedded, qualitative evaluation of the Collaborative Working Group (CWG) process, a structured approach we developed to iteratively refine a complex intervention prior to a randomised controlled trial.

**Methods:**

The CWG was designed and delivered to support iterative refinements to a complex intervention pre-trial as part of the DAFNEplus research programme, a large intervention development study to refine and pilot a self-management education programme for people with type 1 diabetes. The CWG comprised monthly teleconferences and four strategically timed face-to-face meetings throughout the pre-trial period to support knowledge sharing between the practitioners delivering the pilot intervention and the researchers evaluating it. We conducted an embedded qualitative study to elicit CWG members’ experiences and to hear their views of the acceptability, feasibility and effectiveness of the approach. Data were generated through two focus groups with CWG members, four individual interviews with CWG facilitators and documentary analysis of meeting materials.

**Results:**

This qualitative evaluation shows that participants generally found the CWG to be an acceptable, feasible and useful approach to supporting complex intervention refinement pre-trial. The qualitative findings highlight five critical elements that shape the success and acceptability of the CWG approach: funnelling knowledge over time, negotiating trust, balancing practicalities, making epistemic compromises and managing power and hierarchy in decision-making. The findings highlight the need to build in adequate time and resources to support trust-building and knowledge sharing throughout each stage in the research process, in addition to the benefits of creating boundary-spanning roles.

**Conclusions:**

This paper showcases a practical approach to operationalising collaborative intervention refinement and development pre-trial, with tangible lessons and recommendations for future research teams. The paper adds new insights and practical guidance to the intervention development and knowledge mobilisation fields.

**Supplementary Information:**

The online version contains supplementary material available at 10.1186/s40814-024-01576-3.

## Key messages regarding feasibility

- What uncertainties existed regarding feasibility?

The literature on developing complex interventions focuses on the theory and principles behind high-quality intervention development, with less explicit attention given to the practical aspects of how this should be achieved.

- What are the key feasibility findings?

The CWG method offers a structured approach to collaborative knowledge mobilisation and decision-making in the development and refinement of a complex intervention. Practical recommendations are also made regarding factoring the activities required into the grant application stage.

- What are the implications of the feasibility findings for the design of the main study?

The co-production of the DAFNEplus programme by practitioners, researchers and PPIE, using the CWG method resulted in a robustly developed complex intervention.

## Background

Much of the literature on developing complex interventions focuses on the theory and principles behind high-quality intervention development, with less explicit attention given to the practical aspects of how this should be achieved [1]. For instance, the Medical Research Council (MRC) recommends that pilot studies of complex interventions incorporate process evaluations to assess intervention acceptability, feasibility and fidelity and to refine the intervention before it is delivered at scale in a definitive study [2]. However, neither the original nor updated versions of the MRC guidance [2–4] provide tangible direction for how, or when, to translate the learning from process evaluations (or other recommended pre-trial activities) into the intervention design. Similarly, the INDEX framework [5, 6] outlines a comprehensive set of steps for intervention development but does not offer practical advice for how to use findings from primary data collection in pilot studies. It also fails to incorporate practitioners’ experiences of delivering a pilot intervention, to refine a complex intervention prior to a trial.

In light of this gap in guidance, whilst undertaking a large complex intervention development study in 2017–2018, we developed the “Collaborative Working Group” (CWG) method [7]. The CWG is a structured approach to collaborative decision-making that supports both the researchers developing and evaluating a complex intervention, and the practitioners delivering it, to work together to share knowledge and iteratively refine the intervention during the pre-trial stage. Thus, we viewed the pilot phase as an opportunity to co-create the intervention by weaving together knowledge generated through the process evaluation with practitioner experience and expertise. We have published the core components of the CWG process and a protocol for its evaluation elsewhere [7]. In this paper, we present findings from the embedded evaluation of the CWG method to learn and share important lessons about the practicalities of pre-trial collaborative intervention refinement. Based on practitioner and researcher views and experiences of participating in the CWG process, we share some of the challenges and opportunities posed by the CWG process and make recommendations for how this might be used and improved in the future.

## Overview of the CWG process

The CWG was designed and delivered as part of a large intervention development study to refine and pilot a self-management education programme for people with type 1 diabetes, called DAFNE*plus*. The aim of the pre-trial work was to develop an intervention adapted from the internationally established Dose Adjustment for Normal Eating (DAFNE): a 5-day structured education programme delivered by dietitians, diabetes specialist nurses and physicians that provides people with the skills to count carbohydrates, adjust their insulin doses and monitor and improve blood glucose management [8]. The intention of the DAFNE*plus* programme grant was to refine and enhance the existing DAFNE intervention through the addition of behaviour change theory, clinical psychological principles, new technology and a programme of structured follow-up support for up to a year post-course [9–12]. The course content from the original DAFNE curriculum was first adapted using a behaviour change model before then being piloted in three National Health Service (NHS) diabetes centres in two waves. We conducted a process evaluation alongside the pilots, comprising course observations and interviews with participants immediately post-course, then at three and six months post-course. Refinements were made to each element of the intervention (education course, technology and follow-up support) incrementally between each wave in response to process evaluation data, practitioners’ experience of delivering the intervention and patient and public involvement via an advisory group. As described in the protocol paper [7], the CWG also consulted regularly with the DAFNE*plus* patient and public involvement (PPI) group programme, who met regularly to discuss all aspects of the programme grant and provide expert advice on issues and questions raised by the CWG throughout intervention refinement.

The CWG facilitated a process of iterative intervention refinement through a combination of monthly teleconferences and four face-to-face workshops between June 2017 to June 2018. The aim, structure and process for these meetings are detailed in our protocol paper [7]. To summarise, the teleconferences enabled practitioners to share regular reflections on intervention delivery and researchers to share emerging findings from the ongoing process evaluations. The CWG chair [JPB] invited members to contribute content via email approximately one week in advance of each teleconference and then synthesised the responses into a ‘what’, ‘so what’, ‘now what’ matrix (see Additional file 1). The matrix provided the structure for each meeting, and the chair facilitated discussion of each row and column. The matrix operated as a dynamic document, with the CWG chair making notes in the matrix to capture discussion points and actions during the meeting so it could be shared immediately or as soon as possible after each meeting. As the purpose of the teleconferences was predominantly about sharing information, they typically did not result in immediate changes to the intervention but in identifying what more needed to be known before contemplating making refinements. Typically, this involved actions for practitioners to reflect further as they delivered the pilot intervention in their different contexts, or actions for the research team to add new or different questions to process evaluation interview topic guides.

The purpose of the face-to-face meetings was to reflect on all knowledge to date and to agree upon definitive changes to the intervention. Four full-day face-to-face meetings took place; two meetings between waves 1 and 2, and two meetings between wave 2 and the definitive trial (see Fig. 2 in our protocol paper [7] for more detail). Prior to each face-to-face meeting, the CWG chair and co-chair [JPB and EC] gathered and combined the different knowledge sources feeding into CWG decision-making. This involved bringing together up-to-date findings from the ongoing analysis of process evaluation data, all the content from interim CWG teleconference matrices and the minutes from the DAFNE*plus* PPI advisory group meetings. This involved processing a large amount of information and converting it into an accessible and manageable format to support focussed discussion and decision-making. The CWG chairs worked together (sometimes bringing in other members of the CWG team) to synthesise all this information into a series of ‘what’ ‘so what’ ‘now what’ matrices organised under different inductively derived themes. At the face-to-face meetings, CWG members were divided into small working groups to discuss each theme: they were asked to reflect on the knowledge summaries under the ‘what’ column in each matrix and to log their decisions in the ‘now what’ column. In advance of the meeting, the CWG chairs provided discussion prompt questions in the ‘so what’ column with the aim of facilitating focussed and in-depth discussion. The CWG chairs planned to ensure that each of the small working groups contained a mix of practitioners and researchers and that key decision makers were involved in each themed discussion (for example, that a dietitian was present in any discussion of carbohydrate counting, or that a behavioural psychologist was present in any discussion of patient action plans). Final matrices were prepared in the days preceding face-to-face meetings to maximise timeliness and ensure that discussions focussed on the most up-to-date information. This meant that CWG members did not have access to the information beforehand. The CWG chairs produced a report from each face-to-face meeting within one week, which provided a summary overview of decisions made alongside copies of each of the thematic decision matrices. This report was colour coded according to which member of the CWG was responsible for actioning a change, and many of the actions involved further small group work outside face-to-face meetings to deliver the work agreed.

## Methods

We conducted an embedded qualitative study of the CWG method used within the DAFNE*plus* programme, eliciting CWG members’ opinions about the acceptability, feasibility and effectiveness of the CWG process. The aim of this study was to understand how the CWG works and how its processes and procedures might be improved for use in future pre-trial pilots and intervention development studies. We addressed the following research questions, from the perspectives of CWG members:

1. How did the CWG facilitate decisions about intervention refinement?
2. What were the opportunities and challenges posed by the CWG process?
3. What were researchers’ and practitioners’ experiences of, and views about, taking part in the CWG process?
4. How could the CWG process be improved?

### Participants and recruitment

All members (*n* = 25) of the DAFNE*plus* programme grant team participating in the CWG teleconferences and face-to-face meetings were invited to take part in the embedded evaluation study. JPB sent a study invitation and participant information sheet via email and gave potential participants the opportunity to address any questions before returning a signed consent sheet. Participants were asked to provide blanket consent to all aspects of the study, in the knowledge that they could withdraw from the study, or any specific aspect of it, at any time. All 25 members of the CWG consented to taking part in the qualitative evaluation. Table 1 provides a breakdown of participants and their involvement in the study.

**Table 1:** Membership and roles of the CWG

Category	*N*	Role
Practitioners delivering intervention	12	Intervention design and delivery—led by DAFNE-trained dietitian and nurse educators, with input from DAFNE-trained physicians (3 NHS centres)
* Diabetes Specialist Dietitians*	*4*	
* Diabetes Specialist Nurses*	*5*	
* Consultant Diabetologists *	*3*	
Clinical Psychologists	2	Intervention design and provision of training and supervision to DAFNE practitioners delivering the intervention
Process evaluation research teams	6	Behavioural psychologists: intervention design and process evaluation, via post-course participant and practitioner interviews, observation and fidelity assessment Social scientists: process evaluation via longitudinal interviews at 3 and 6 months
* Behavioural psychologists*	*4*	
* Social scientists*	*3*^a^	
Study Manager	2^b^	Oversight of DAFNE*plus* research programme (project management, ethics and governance, etc.) with expertise in knowledge mobilisation and shared facilitation of face-to-face meetings
CWG Chair	1	Responsible for chairing the CWG meetings and delivery of CWG processes. Shared facilitation of face-to-face meetings with Study Manager. Member of the social science process evaluation team with expertise in knowledge mobilisation
CWG Administrator	1	Administrative support to CWG meetings (minute taking; room booking; organisation of meetings and travel)
Chief Investigator	1	Leadership of DAFNE*plus* Programme Grant and research active Consultant Diabetologist
**Total**	**25**	

^a^One of the social scientists was also the CWG chair. They are included in both sections of the table to highlight the dual role but are only counted once in the final total

^b^A new Study Manager was appointed in March 2018, but the previous incumbent maintained their role as the co-chair in the CWG

### Data collection

Data were collected longitudinally using a combination of methods: focus groups with CWG members; repeat individual interviews with the CWG chair [JPB] and DAFNE*plus* study manager/CWG co-chair [EC]; reflective field notes written by the CWG chair; and materials generated as part of the CWG process. As the primary focus of this study was eliciting members’ experiences of, and opinions about the CWG, focus group data was the primary data source for our analysis. We conducted two focus groups at the end of the second and fourth face-to-face CWG meetings, lasting 48 and 55 min, respectively. The format, number and scheduling of CWG meetings are shown in Fig. 2 in the protocol paper [7]. Data collection was timed purposely to ensure that CWG members had sufficient experience of the CWG process to inform their opinions in the first focus group, whilst the second focus group allowed both for insights into final intervention decisions and for reflection on the entire CWG journey. The focus groups were facilitated by an independent researcher [RGW] to enable participants to share their opinions more freely. Each focus group was guided flexibly using a semi-structured topic guide (see Additional file 2), which explored participants’ views about the frequency, method, timing and impact of the meetings and how, if at all, communication and collaborative working could be improved in the future. To identify and explore how the CWG facilitated tangible decisions about intervention refinement, we also asked participants to identify their key moments in the process and to describe these in detail.

The same independent researcher also conducted individual telephone interviews with the CWG chair [JPB] and the DAFNE*plus* study manager/CWG co-chair [EC] in the week after the second and fourth face-to-face meetings. These interviews lasted between 40 and 95 min and were guided using a semi-structured topic guide covering the same questions as the focus groups. To provide additional data and support researcher reflexivity, the first author and chair of the CWG [JPB] kept a diary, capturing regular reflections about what worked well and not so well during each meeting. They reflected on the content of these notes in their individual interviews. We also audio-recorded all CWG teleconference and face-to-face discussions and kept all materials (i.e., meeting documentation and emails related to the CWG) to provide a further source of data.

### Data analysis

Focus groups and interviews were digitally audio-recorded, transcribed verbatim and analysed thematically, guided by Braun and Clarke [13] and Terry et al. [14]. We analysed the transcripts inductively, whilst being mindful of our original research questions. This involved a familiarisation stage where three researchers [RGW, JPB, EC] immersed themselves in all transcripts in detail and made notes about their initial impressions and ideas for themes. We then coded the whole dataset independently, writing inductively generated codes in the margins of transcripts, before coming together as a group over a series of telephone meetings to collectively refine our multiple codes into a set of broad themes. In keeping with a reflexive approach to thematic analysis, the purpose of coding and retrieving the data independently was to maintain openness and bring multiple perspectives to theme generation [15]. Two researchers [JBP and EC] then returned to the transcripts and independently coded all data into the five broad themes we had identified, using NVivo 12 to further enhance understanding and check that the themes accounted for the whole dataset. This process helped ensure that we stayed close to the data and had not taken our analysis too far away from participants’ stories and perspectives [14]. This was especially important in our study because JPB and EC were analysing the data as key members of the CWG process, and this level of rigour was essential to transparency, accountability and reflexivity. All three researchers were involved in the process of finalising the themes by reviewing and discussing areas of overlap in the data and agreeing the discrete definition, scope and content of each theme. We also explored if, and how, participants’ views about the CWG changed over time by comparing responses in the first and second focus groups and interviews. Following interview and focus group analysis, JPB then reviewed all teleconference matrices and face-to-face meeting documentation to identify and explore illustrative examples of the CWG decision-making process. JPB identified illustrations and examples of the themes identified in the interview data, in addition to remaining open to other inductively derived ideas, which allowed us to confirm and expand issues arising in the interview and focus groups by triangulating findings across different data sources.

### Ethical considerations

Ethical approval was granted by the Usher Research Ethics Group at the University of Edinburgh (Ref 1732). The promise of full anonymity was not possible, owing to the small number of CWG members and their distinct professional roles; therefore, participants were asked to give their consent with this in mind, and all were given the opportunity to review this paper prior to submission, with the option to request rewording or removal of text relating to themselves if necessary. This process also enabled CWG members to provide feedback on the interpretation and presentation of findings and to shape the analysis and write up of the paper. In our findings, we have chosen to identify participants using the broad labels of either DAFNE*plus* ‘researcher’ or ‘practitioner’ to preserve anonymity. In addition, we have explicitly labelled quotations from the ‘CWG chair’ and ‘CWG co-chair’ to ensure transparency and promote trustworthiness in our analysis and presentation of results.

## Results

There was consensus amongst participants that the CWG was generally effective in enabling the researchers evaluating the pilot DAFNE*plus* intervention, and the practitioners delivering it, to work together to refine the intervention pre-trial:

I think it has been essential in bringing different disciplines together to work in a collaborative way, which I’m not sure how it would have worked without this group (Researcher FG2)

I think it has been crucial here, I don’t think it could have achieved it without it (Practitioner, FG2)

it’s been very educational in an academic sense to see how this can be done and particularly the way the different disciplines have come together to do exactly what it says on the tin, so I’ve been extremely impressed… If I was going to write another grant… then I would put this [as a] work package for sure, and I think that’s quite telling isn’t it really (Researcher FG2)

Where participants were less enthusiastic about the CWG initially, these opinions shifted towards the end of the process as they reflected on its usefulness in refining the intervention, for example:

In the beginning I would have said not, definitely would not recommend. But honestly, now it seems to have worked out and I wouldn’t be able to suggest an alternative (Researcher FG2)

So, given that the CWG appears to have facilitated an acceptable approach to interdisciplinary collaboration, our data analysis sought to understand how this happened and the conditions required for achieving effective collaboration. We generated five themes, albeit with some overlap, that explain how the CWG impacted on intervention refinement and influenced participants’ experiences of the process. In summary, the critical elements identified of the CWG approach relate to:

1. Funnelling knowledge over time
2. Negotiating trust
3. Balancing the practicalities
4. Making epistemic compromises
5. Managing power and hierarchy in decision-making

### Funnelling knowledge over time

The funnelling process was intentionally built into the CWG in the form of the ‘What? So What? Now What’ matrix described above and previously [7]. The matrix supported the group in:

Moving through that process from description, to discussion, to decisions (CWG Chair Int 1)

Across both focus groups, there was consensus that the CWG matrix provided a useful decision-making structure:

I think that is actually a very effective way of thinking about the data and how to use it for the next steps particularly because we are so iterative at the moment, it does really mean that we are moving very fast from looking at something to actually making a definitive plan for actioning it (Practitioner FG1)

Although each meeting was guided by the same format, its focus shifted over time according to the stage of intervention refinement. At the beginning, the ‘What’ and ‘So What’ questions were intended to open up multiple possibilities whilst the ‘Now What’ question was used to identify what additional information was needed to enable future decisions. This level of openness and the slower pace during initial meetings could be frustrating for practitioners who wanted to action changes:

So, when we were first having the telephone calls, I found them a little bit frustrating and I wasn’t quite sure where we were going, or what was really the point of it… it just became a bit of a description of people’s experiences as they were having them (Practitioner, FG1)

For the researchers, however, this approach was considered essential for refining interview schedules and enabling them to collect the most useful data to inform decision-making:

The CWG was actually really effective in flagging that up early on so that we could collect the right data in response to what was happening in practice (CWG Chair Int 2)

With hindsight, practitioners in the second focus group also described appreciating the usefulness of the early teleconferences:

When we look back, a lot of lightbulb moments have happened really in those telephone conversations and sort of just starting to highlight which bits we need to go away and look at (Practitioner FG2)

Participants also reflected on the importance of funnelling knowledge over time to support decision-making rather than making ad hoc refinements to the intervention before there was sufficient evidence from more than one source to warrant change:

Today we’re talking about issues that we’ve known are a bit of a thing for quite some time, but it wasn’t the right time to discuss it or bring it up… I’ve been banging on about [one possible change to the intervention] for months but now we’ve got data and actually now it’s in the CWG process it becomes a thing. It’s not just one person’s opinion you know so actually that’s very powerful (Practitioner FG1)

As the CWG meetings progressed, the matrices were seen as becoming more useful in ensuring that CWG members made pragmatic decisions, balancing the need to refine the intervention with the available capacity, resources and time:

Parts of the CWG process have been quite good to tether us to what’s reasonable, what we can do in the time, rather than just having a wish list. So that’s been quite helpful actually. (Practitioner FG1)

As well as funnelling knowledge over time, participants felt that the CWG approach also became more streamlined and effective. The repetition of the ‘What? So What? Now What?’ matrix in every meeting meant that these prompt questions became ingrained and habitual in CWG members’ thoughts and discussions:

Maybe the ‘so what’ changes over time, it’s like more useful at the beginning… so we are better at interpreting the relevance of the source data maybe by this stage, so we don’t need handholding around so much (Practitioner FG2)

I think as people got a lot more familiar with it, they probably started pre-empting some of the questions that were in there, or they changed the information they were giving me, so it was much more specific and what was required for the matrices as we went along (CWG Chair Int 1)

As a core function of the CWG method, funnelling knowledge over time ensures that decisions about intervention refinement are based on sufficient evidence and consider pragmatic concerns. There are two illustrative examples of how the CWG supported decision-making over time in Tables 2 and 3.

**Table 2:** Illustrative example of the CWG in action: clarifying roles and expectations for technology-assisted self-monitoring

• A key element of the DAFNE*plus* intervention was to give people the skills and means to regularly self-monitor and review their blood glucose (BG) readings, to identify patterns and make necessary adjustments to optimise their BG management. To support this, DAFNE*plus* participants were given new technology that enabled them to upload their BG readings to a website where they could review their own data • In the initial CWG teleconferences, practitioners delivering the wave 1 intervention in each of the pilot sites highlighted that there was a lack of engagement with the technology. Practitioners reported that this was partly due to technical reasons, which the CWG was able to feedback to the engineers who promptly resolved data upload and connectivity issues • At subsequent CWG teleconferences, however, practitioners were continuing to report low engagement with the technology, and even where participants were uploading their BG readings, they were not necessarily then reviewing their data online. A decision was taken at a teleconference to adapt the topic guide for the post-intervention qualitative interviews as part of the process evaluation to explore participants’ understandings of the technology, their perceived barriers to its use and what their expectations were around data monitoring. Analysis of the interview data found that many participants believed that the online data was being reviewed regularly by their practitioner, who would then advise them what adjustments to make (which is counter to the self-management function that was intended) • At a face-to-face CWG meeting, decisions were therefore taken to ensure *DAFNEplus* participants received clearer messaging about the self-management purpose of the technology; to set up an automated reminders to upload and review data; to create a ‘flag’ system that would alert participants to potentially concerning patterns in their data • These actions were taken forward by a small working group outside of the CWG process and their proposed solutions reviewed by the study Patient Advisory Group. The Patient Advisory Group provided suggestions for communicating expectations more clearly and suggested that the automated messages should be termed ‘amber flags’ that give participants the option to click ‘I am dealing with this myself’ or ‘I would like support’, whereupon they are contacted by a practitioner • The CWG process enabled early identification of this issue and provided structured opportunities to generate and combine knowledge from a range of sources (practitioner feedback, qualitative data, PPI feedback) to inform refinements to the intervention which could then be tested out and fine-tuned in Wave 2 courses

**Table 3:** Illustrative example of the CWG in action: revising the action planning element ofDAFNE*plus*

• Enabling participants to set their own action plans was a central element of the self-management, behaviour change approach underpinning *DAFNEplus* • In the first CWG teleconference, practitioners in the first site delivering Wave 1 courses shared that they did not feel that action plans were working well. Rather than make any changes at this early stage, the CWG waited to hear feedback from the other two pilot sites who had still to deliver their first course • In the following CWG teleconferences, practitioner feedback from other sites, combined with data from the observations undertaken as part of the process evaluation, confirmed some issues with the action planning process. For example, that there was not enough time at the end of the day to complete the action plan forms, as well as practitioners feeling that the forms were too numerous and complex • Prompted by these discussions at the teleconferences, adaptations were made to the qualitative interview topic guide for the post-course interviews with both practitioners and participants to explore action planning in more detail. Practitioner interviews highlighted that they lacked some confidence in action planning, found it difficult to provide 1:1 support in the short time allocated for it, and felt that the formality of ‘signing off’ participants’ actions plans was counter-intuitive to the self- management ethos. Interviews with DAFNE*plus* participants identified that they did not like completing action plans at the end of each day on the course, as they felt tired and found it hard to recall specific behaviour changes they had identified earlier. They also had mixed opinions about signing their action plans; whilst some felt it was patronising, others felt motivated by the level of accountability it created • Combining this data in small group discussions at a face-to-face CWG meeting, the group took the following decisions: the action planning session would take place at varied times throughout the day; participants would be invited to sign their plans but this was not mandatory; practitioners would not ‘sign off’ action plans so that they belonged to participants as aligned with self-management • These changes were adopted in wave 2, with continued attention given to action planning in process evaluation interviews • The Patient Advisory Group were also asked to review the action planning documentation and suggested using a single open-structured action plan template, rather than having different action planning templates for specific behaviours • The combined feedback from practitioners, DAFNE*plus* participants and PPI representatives was discussed at the next face-to-face CWG meeting, resulting in a decision to move to open templates for action plans and enhancing practitioner training on action planning to help them feel more confident using this approach • Follow-up interviews with DAFNE*plus* participants at three months post-course in Wave 2 generated much more positive data around the use of action plans, demonstrating that participants and practitioners had a clearer understanding of their purpose, allowing the action planning approach to be confirmed pre-trial in an iterative, evidence-informed way	

### Negotiating trust

Practitioners and researchers alike spoke of the importance of having confidence and trust in the CWG process, and in the other CWG members and chairs. As a new process that involved both researchers and practitioners stepping outside their usual ways of doing things, participants felt that trust was not immediate and needed time to develop. Researchers who were used to sharing their findings only after data analysis was complete, described feeling uncomfortable when asked to share their analytic ideas in real-time. Involving practitioners so directly in the analysis process could feel alien and, especially in the early stages, researchers felt uneasy about trusting the other group members with their data:

I think that’s the thing because it takes the time to generate the data, when you have spent that much time looking at things in detail … you become attached to those things. And then when other people come, because that’s the process, and make decisions about what goes in [to the intervention] and what doesn’t, it’s inevitable you know, I don’t want to bang on about it, but things do fall off the side, that’s been a difficult thing for us (Researcher, FG1)

Practitioners similarly described being wary of sharing their experiential reflections in the early stages of the pilot intervention because they worried that CWG group might over-amplify their preliminary insights, resulting in unnecessarily premature decisions to change the intervention:

We haven’t had a lot of experience as educators running these courses and so the things we were sending through were kind of sort of quite individual remarks (Practitioner, FG1)

Over time, however, CWG members’ trust in one another, in the CWG chair and co-chair, and in the CWG process appeared to have strengthened and grown, with a discernible shift in mood reported.

As we’ve got to know each other and understood our various strengths, and indeed weaknesses, it became easier to integrate, and I think this process facilitated that. (Practitioner, FG2)

Part of the CWG process, which maybe we wouldn’t have expected to get out of it, is this idea of trusting that things fall away and come back into the frame. If they are important enough, they probably will. Otherwise you are trying to hold onto everything and it’s impossible, so there’s something about trusting the organic process of the CWG and that it would raise what’s important in a timely way, which maybe we weren’t aware that process was going on (Practitioner, FG2)

The CWG chairs anticipated that it would take time for group members to embrace a new process and sought to engender trust from the outset by consulting with prospective CWG members when designing the approach. Whilst this was considered important groundwork, the CWG chair also reflected that trust needed to accumulate over time as part of the process:

In order for people to invest in something, they needed to be convinced that it’s working. They needed to see evidence of it working in order to be motivated to keep coming along and I think that’s happening (CWG Chair, Int1)

Indeed, trust in the CWG group and in the decision-making process became so well-established that participants said they felt it was difficult to achieve closure before transitioning into the main trial:

People were nervous I think about the formality of it initially… but everybody seems to have relaxed into it and almost to the point where we can’t stop them from wanting to change the intervention…We took ages to get them into it and now we want them to stop and it’s quite hard! (CWG Co-chair, Int2)

Negotiating trust in each other and in the process was therefore perceived to be critical to the success of the CWG.

### Balancing the practicalities

CWG members reflected on the need to achieve a balance regarding the frequency and format of the meetings, adequate resourcing and the scale of the task involved. There were mixed opinions on the frequency of the monthly meetings, with concerns about the time commitment required. However, members were generally cognisant of the need to meet regularly to maintain momentum to enable real-time communication. It could be difficult to schedule meetings at an optimal time for all members, particularly practitioners with busy clinical commitments and in some cases, part-time working patterns to accommodate. As a result, there was inconsistent representation from practitioners across the three DAFNE*plus* pilot sites in the CWG, something that was highlighted both by group members and chairs:

We’d have to ask the people who aren’t here (Practitioner FG2)

We’ve had varying commitments and I think it’s because people are so busy and overstretched and the situation in the NHS as it as at the moment… it’s a bit of a concern because you know, I think it is really important that everyone’s knowledge is valued and everyone has a chance to input when we are making decisions based on the collective experience (CWG Chair Int 1)

Regarding the format of meetings, group members grew to appreciate the combination of face-to-face whole-day meetings and the 90-min teleconferences, which served different but complementary purposes:

You need that combination of the phone and the face-to-face. I think the face to face has been important for building up relationships and understanding what different roles [are] in the process. (Practitioner, FG2)

I think both worked well. And I think the phone worked surprisingly well and there’s definitely instances where we made huge steps forward in some of the phone meetings, when using the face-to-face meetings for more chewy issues. (Practitioner, FG2)

CWG members also commented on the volume of work undertaken by the CWG chairs in advance of each meeting, and in particular the face-to-face events. The scale of the task was acknowledged, and participants were keen to highlight the need to include adequate costings for any future grants involving an intervention development phase:

There’s so much preparation that went in to making it easier to contribute and partake. Like the volume of the data and then picking out what’s useful, what can realistically be discussed, how can we divide it into topics, how can we then divide it into groups, how can we divide it into meaningful groups, how can we divide up days so we can have pragmatic steps (Researcher, FG1)

It feels like an important lesson for me… to make sure there are more people who are costed in for more time to really put the effort into this intervention refinement phase (CWG Chair Int 1)

### Making epistemic compromises

Combining health and social science researchers, clinical psychologists, physicians, dietitians, nurses, behavioural scientists and technologists, the CWG brought together members with different epistemological viewpoints. Each held different assumptions about what counted as knowledge, and what type of knowledge should inform intervention refinement and how.

We weren’t going in with recommendations for what should happen, we were going in with a high-level summary of the what the data told us. Essentially, we were populating the What? column and then we were engaged as equals in these small groups [doing] the next layer of data analysis… practitioners and researchers working together to consider what does this potentially mean and now what are we going to do about it (CWG Chair Int 1)

However, researchers initially raised concerns about whether and how the integrity of their data and analytic insights were preserved in the CWG matrices:

This almost didn’t look like qualitative data at all, it was so pared down…it’s almost like managing your expectations of different qualitative data and different kinds of analysis, whether this is good enough (Researcher FG2)

In contrast, practitioners often questioned whether their experiential and professional knowledge was legitimate enough to share at CWG meetings:

It might have just been particular to that group of patients or you know a strong individual in the group and then it’s difficult to weight that information in terms of what’s important… (Practitioner, FG1)

You know what would be considered important clinically might not be considered important on a kind of other disciplines point of view or a pure science point of view (Practitioner, FG1)

By the second focus group, however, participants noted greater confidence in the combination of different knowledge types as being equally valuable in shaping intervention refinement. Researchers appeared to have shifted their perspectives on hierarchies of evidence and the quality of the material prepared for the CWG meetings:

I think in the beginning we were really worried that this was data analysis and it wasn’t rigorous… but I think we’ve kind of realised that it’s one strand and… to be more pragmatic (Researcher FG2)

I felt much more confident about what was coming out because there was a diversity of perspectives that we don’t often get when we do our qualitative reporting… [you come] up with the sort of wish list of, you know, the perfect scenarios… and it’s really helpful having the grounding in reality that this has to be implementable, cost effective you know, it just means you can do something a lot more responsible and useful with qualitative data than qualitative researchers alone can do (Researcher, FG2)

By contrast, practitioners came to understand the reciprocal and complementary relationship between practice expertise and research evidence, rather than one being more legitimate or weightier than the other:

Sometimes the way the data is presented, it’s acontextual and that might lead to different conclusions, but that’s the great thing about this process is that we are able to integrate… the clinical part of things (Practitioner FG2)

As well as a growing trust in one another and in the process, participants reflected that epistemic compromise was enabled by having a boundary-spanning chair (who is both a researcher and healthcare professional):

[The CWG chair] is a practitioner and she’s a researcher. And you can tell that she is comfortable in that divide between the two and I think […] maybe that’s why it works so well (CWG Co-chair, Int1)

I was thinking about it today and just having someone independent take all of the different streams, all of different sources of data and pull it into one thing just enables us to, it just decentralises your own perspective of things and allows you a more rounded view of the task at hand (Practitioner, FG2)

The universality of the CWG ‘What? So What? Now What’ matrix, which underpinned the structure of every meeting, also helped to support inter- and transdisciplinary conversations:

I think it’s something that is good for communicating across different groups so it’s like, I feel like it does work in a language that everybody can understand, so that’s not just between the researchers and practitioners, but even between the different disciplines (CWG Co-chair, Int1)

### Managing power and hierarchy in decision-making

The CWG process was designed as a knowledge-sharing platform with a flattened hierarchy:

How the process came about was that I kind of drafted this strategy for mobilising knowledge in the pre-trial phase of DAFNE*plus* and in writing that I had put in a big section about the ethos of collaborative working and valuing different types of knowledge and really describing the range of knowledge and the non-hierarchical approach we were taking to knowledge (CWG Chair, Int1)

Although established with this intention, participants reflected that existing power relations within the study team (many of whom had worked together in previous studies) played out during early CWG meetings. At the first focus group, several members said they were concerned about how issues of power and hierarchy would impact on the quality of decision-making:

Yeah, I mean I think as an interpersonal process there are clear issues of power and hierarchy in this process that I think made some voices have more of a say and some voices that were kind of less listened to. (Practitioner, FG1)

The power dynamic also manifested in the interplay between participants working on different workstreams in the wider DAFNE*plus* grant. Before the CWG was established, participants suggested that each workstream was somewhat siloed and the CWG brought a welcome opportunity for integration. However, throughout the process, different team members said they still felt they needed to vie for attention to their own components, with a fear that certain elements were given less priority than others. Over time, however, as CWG members compromised to accommodate other viewpoints, they began to decentralise their own component, instead seeing it as one part of a bigger integrated intervention:

I think it’s felt like today we have, this is the first, not the first, but this was a really useful CWG for the technology. So, and we probably wouldn’t have got there if we hadn’t done the CWG process about other things. So, I think you just have to accept that it’s not always going to be maximally relevant for every single thing and you just have to tolerate that, that it’s not perfect and it might feel irritating at times (Practitioner, FG2)

A key enabler to reducing power imbalances over time was gathering and acting upon informal feedback about the CWG process as the work progressed. For example, after the first face-to-face meeting, participants suggested that it might be helpful to reconfigure the small working groups to ensure that people with the right experience and expertise were discussing the most appropriate topics. The CWG chair and co-chair made this change and continued to seek feedback on the CWG process and respond to participant questions and concerns. This was noted by participants in the second focus group as having a positive effect on building trust in the process:

So, I think the process of doing it encouraged and engendered trust and led to a reduction in power imbalances that led to a more productive process. And I think that’s not just from, it’s not just the people round this table, I think it’s an interaction between the people running the process as well. So, I think it was quite a bidirectional trust, kind of developed over time. (Practitioner, FG2)

With growing trust and increasing compromise and collaboration, participant views seemed to have shifted by the second focus group, demonstrating a sharp contrast in perspectives:

Over time it grew to a much more egalitarian approach where I think everybody’s views were more equally represented, I think it was always equal in representation. (Researcher, FG2)

As another CWG member reflected:

…it helps seeing the process through, so seeing the work we did early on be useful and make a difference to improve the intervention exactly how we intended it to. I guess that helps ease any concerns about power and balance because you see that it is actually working (Researcher, FG2)

## Discussion

The CWG method is a structured approach to collaborative decision-making that mobilises knowledge between researchers and practitioners to iteratively refine a complex intervention pre-trial. This qualitative evaluation shows that participants generally found the CWG to be an acceptable, feasible and useful approach. Rather than bookending knowledge exchange activities at the beginning and end of the research process, the CWG was perceived to be successful in embedding collaborative knowledge sharing throughout the DAFNE*plus* pilot study. A comparative ethnography of three large scale, applied implementation research projects [16] identified few similar instances of knowledge brokering between core research teams and stakeholders during active data collection and analysis. Our work therefore adds new insights and practical guidance to intervention development and knowledge mobilisation fields. Whereas our first paper [7] outlined the practical steps in setting up the CWG, this paper reflects on the conditions required to make the CWG work effectively to enable intervention development and refinement. Based on listening to the perspectives and experiences of CWG members, our findings suggest that there are five critical elements that shape the success and acceptability of the CWG approach: funnelling knowledge over time, negotiating trust, balancing practicalities, making epistemic compromises and managing power and hierarchy in decision-making.

Looking to the broader knowledge mobilisation literature, the CWG and accompanying decision matrix (‘what’, ‘so what’, ‘now what’) could be conceptualised as a ‘boundary object.’ As defined by Carlile [17], boundary objects provide ‘a concrete means for individuals to specify and learn about their differences and dependencies across a given boundary’ (p.452). It is widely recognised that researchers and practitioners are separated by different boundaries, including geographical, temporal, cultural, organisational and professional domains [18]. Each of the CWG members was bound to different social and professional identities that shaped their perceptions of what knowledge was most relevant, credible and valuable in refining the DAFNE*plus* intervention. Reflecting on our data in relation to trust, power and epistemic compromise, our findings evidence that the CWG method supported practitioners and researchers to navigate the three layers of knowledge boundaries identified by Carlile [17]: syntactic (where participants recognise their differences and develop ways of talking to each other to sufficiently transfer knowledge from one discipline to another); semantic (where participants negotiate shared meanings and translate learning between disciplines); and pragmatic (where participants’ combined knowledge is transformed into new collective understandings that transcend uni-disciplinary boundaries).

The CWG method was purposefully designed with an inbuilt progressive structure to funnel knowledge over time and guide participants from multi- to inter- to transdisciplinary conversations. Siedlok and Hibbert [19] define transdisciplinarity as a ‘fusion of disciplines through a focus on irreducibly complex problems… [resulting in] coherence, unity and simplicity of knowledge’ (p.198). This is in contrast to both multi-disciplinary approaches, which involve divergent disciplines working towards separate but aligned goals using their own disciplinary understandings, and interdisciplinary approaches, which involve bidirectional knowledge sharing to achieve shared goals [19]. The CWG method started by first valuing participants’ professional and subgroup identities (e.g., researcher or practitioner), before employing structured, collective, knowledge-sharing activities that guided participants towards a superordinate identity around a singular shared goal. Early meetings provided space to focus on individual and uni-disciplinary perspectives, as participants shared ‘what’ they were learning in relation to their own elements of the intervention. As the CWG progressed, participants were progressively challenged to take on others’ perspectives and arrive at shared decisions both about their own areas of research and practice, as well as other aspects of the intervention for which they did not have direct responsibility. By the final meetings, participants reported greater cohesion and sense of ownership over the whole intervention, rather than simply the distinct component parts related foremost to their disciplinary expertise.

The progressive funnelling of knowledge over time, gradual negotiation of trust and increasing tolerance for epistemic compromise identified within the CWG bear similarities to the tentative ‘maturation model’ of knowledge brokering proposed by Waring and colleagues [16]. They suggest that brokering knowledge across disciplinary boundaries involves moving through incremental layers: establishing relationships and opportunities for knowledge exchange; transferring knowledge between communities using a common lexicon; problem solving using shared ideas; and establishing a common agenda between aligned communities. Waring et al. have called for further empirical development of their model and we offer our evaluation of the CWG as a practical insight into knowledge brokering ‘in action’, defining the conditions in which collaborative relationships and interventions mature over time. The CWG is both a practical tool (the matrix provided a tangible and consistent tool for shared communication and information sharing) and an epistemic process (the progressively structured process enabled participants to transform their current knowledge into new collective understandings that were implemented in practice). Of course, as Carlile [17] points out, what ‘can be an effective communication tool in one meeting, [is] then a ‘bludgeoning tool’ in the next’ (p452). Thus, we recommend that teams contemplating using the CWG process think carefully about the usefulness of the process and decision-making matrix, adapting it for their own context. This will involve careful reflection on the composition of the team, the trust, power and epistemic dynamics between individuals and disciplines, and the practicalities and resources at their disposal.

Our findings highlight that the CWG method benefits from appropriate chairing and facilitation. Operating in the boundaries between disciplines, personalities and priorities requires the confidence to tolerate and manage tension, confusion, uncertainty and complexity. Some knowledge mobilisation scholars [20, 21] have tried to identify the core characteristics required for such knowledge broker roles, such as organisational ability, effective communication skills and analytical mindedness. Others have highlighted the value in appointing someone in a hybrid role to best enable knowledge brokering (i.e. a person who is both a practitioner *and* a researcher like the CWG chair in our study) because they are best placed politically, relationally and reputationally to sit within and across boundaries [22]. This certainly resonates with our findings, and we suggest that teams planning to use the CWG method in the future consider the qualities, capacity and capability of the person leading this process. That being said, and as Kislov et al. [23] later suggest, it is highly unlikely that one individual will possess all of these qualities and characteristics alone. Waring et al. [18] also suggest that effective knowledge brokering relies less heavily on an individual knowledge broker (such as the CWG chair) but on the mature relationships and understandings formed between knowledge communities themselves. We would similarly propose that the CWG process is by its very nature intended to be collaborative and as hierarchically neutral as possible, and we would rather suggest that the effectiveness of the CWG is not solely reliant upon the chair, but on the creation of effective processes, structures and relationships for collective decision-making.

### Limitations

This study has three main limitations. The first and principal limitation is that this was a self-led evaluation of the approach by the CWG Chair and the Co-Chair (and former Study Manager for DAFNE*plus*). To mitigate the influence of this direct involvement in the delivery and evaluation of the CWG process, an independent researcher undertook all data collection and was involved in the analysis. In addition, in completing data analysis, we tried to rely most heavily upon the focus group accounts but the reporting of this study will be subject to the inescapable influence of our perspectives, as the CWG chairs. Similarly, although we were not present during data collection, participants were aware that the CWG chairs would be analysing the data, which may have hindered a more critical perspective being offered.

The second limitation of this study is the use of focus groups as the main data collection method. Given some of the concerns flagged up about hierarchy and power imbalances, it could be argued that focus groups were not the ideal methods to use, as there is potential for some voices to be ‘heard’ over others. However, this approach was taken on practical grounds to ensure that the data could be collected in a timely and efficient manner, relative to the time-consuming nature of doing individual interviews with busy practitioners and researchers.

The final limitation is that this study is based on only one, very specific case study of developing and refining a complex intervention designed to educate and support adults with type 1 diabetes to promote self-management and reduce diabetes-related complications in the longer term. The group involved in decision-making was particularly large, and this will not always be necessary in other research grants, so some of the design of the CWG is inevitably a function of the context which motivated and hosted it. It would be useful for future research teams to use and evaluate their application of the CWG approach to understand more about the utility and acceptability of the approach. However, we hope that through providing detailed explanations of the approach [7] and evaluating this, other teams can better consider the applicability and practicalities of the CWG approach.

## Recommendations

The development of the CWG approach was borne out of a dearth of directly relevant research literature in this area at the genesis of the work. The since-published INDEX guidance [5, 6] on intervention development does provide a helpful framework for research teams concerned with producing complex health interventions. Yet, whilst this gives much more detail on the actions to be undertaken and that teams must consider the relevance of each action to their particular context, it still lacks detail on some of the more practical aspects of how to deliver those. We therefore hope that the findings from this paper will be helpful to other intervention development teams when planning their intervention development studies: the processes related to stakeholder involvement; team composition and decision-making, refining the intervention and ending the development process. For other research teams wanting to try the CWG approach for intervention development and refinement, we have five recommendations.

1. Explicitly address issues of power, hierarchy and epistemological position from the outset and across the lifespan of the group. In any multi-professional group, these issues will be at play. It is important to engage group members in collaborative reflection that both respects personal and disciplinary expertise whilst also ensuring that all voices are heard to the benefit of the intervention development.
2. Expand the remit of the CWG approach to include more active patient and public participation. As described in the protocol [7], we operated a ‘consultation model’ involving a separate patient advisory group to whom the CWG were accountable. However, we recommend that future applications of the CWG method could explore ways to integrate patient and public perspectives more effectively.
3. Cost sufficient time and resourcing into grant applications to support a CWG process, bearing in mind the time it takes to develop functional trust and build effective working relationships. The efficiency of research teams who collaborate effectively cannot be understated, nor can the challenges of bringing together experts on long-term research grants. In future uses of the CWG approach, attention should be given to fostering trust throughout the beginning, middle and end of the process.
4. Ensure that research teams include members who are comfortable in boundary-spanning roles, or at least have a positive orientation towards applied research to facilitate intervention development. For the CWG to function effectively, all members needed to make compromises regarding their own epistemological viewpoints. Epistemic compromise happened gradually over the course of the CWG process as members grew to trust one another and acknowledged the benefits of working through different interpretations of the data. In DAFNE*plus*, epistemic compromise was facilitated by a chair who spanned both research and practice worlds, and it will be important in future uses of the method to identify avenues for supporting epistemic compromises to occur. More general facilitation skills are also required within intervention development teams.
5. Plan out the practical aspects of the CWG process for intervention development/refinement right from the initial grant application. Working out the most appropriate timing and location of meetings and resourcing is crucial in enabling the CWG to be effective and inclusive. Given that the CWG process was developed and evaluated prior to the Covid-19 pandemic, it is likely that research teams now have considerably more experience in supporting remote collaboration.

Indeed, post-pandemic, several studies have since drawn upon the CWG approach to support the development of healthcare interventions, for example, text messages to promote physical activity after stroke [24] and the implementation of closed-loop technology for pregnant women with type 1 diabetes [25]. Each of these examples illustrates how the approach can be modified for different purposes to enable remote delivery, albeit not without compromise. Rankin et al. [25] applied the approach during a one-off online workshop to help generate meaningful recommendations. Irvine et al. [24] found that the depth of discussion was not always possible during online CWG meetings, so these were supplemented with separate individual meetings to ensure that issues were appropriately addressed.

## Conclusions

This qualitative study describes the practitioner and researcher perspectives of participating in the CWG process. The challenges of traversing disciplines within these spheres and the impact of using ‘what’, ‘so what’, ‘now what’ matrices to funnel the emergent, and different types of evidence and knowledge were evaluated. Adequate resources will be needed to support intervention development when using a CWG process, but the benefits can be substantial. Existing power relationships and the epistemological position of collaborators and stakeholders must be considered and managed collectively to establish new ways of decision-making to avoid following traditional hierarchies. Building trust takes time but is crucial to ensuring different types of evidence are considered with equal weight. Our findings provide practical help to practitioners and researchers planning to design complex interventions during the pre-trial phase.

## Supplementary Information

Additional file 1. Matrix template.

Additional file 2. Topic guide.

## Data Availability

The datasets generated and analysed in the course of this study are not publicly available due to risks to individual privacy. However, they are available, via the corresponding author, on reasonable request.
