# Supplementary material for: The collaborative working group method for pre-trial knowledge mobilisation: a qualitative evaluation of a structured process for iteratively refining a complex intervention (DAFNEplus)
Source: Pilot Feasibility Stud. 2024 Dec 21;10:154. doi: 10.1186/s40814-024-01576-3 (PMC11662412; doi:10.1186/s40814-024-01576-3)
Supplement: Supplementary file 1 — Additional file 1. Matrix template. [file 40814_2024_1576_MOESM1_ESM.docx]

| **WHAT… is going well/ not going as well?** | **SO WHAT… does this mean for**  **(a) intervention delivery and redesign**  **(b) the research process?** | **NOW WHAT… do we recommend needs to be done, by who, when?** |
| --- | --- | --- |
| *COURSE* |  |  |
|  |  |  |
| *FOLLOW UP SUPPORT* |  |  |
|  |  |  |
| *TECHNOLOGY* |  |  |
|  |  |  |
| Notes on completion for the chair | | |
| *Invite CWG members to email feedback one week before the teleconference date. Summarise feedback and populate this column prior to each meeting - condense information into bullet point format so it is quick to read but still thorough and representative. Add as many rows as needed to organise discussion points into broad topics under each of the main intervention components. Prioritise the order in which intervention components are discussed and adapt the matrix structure each month with the highest priority component at the top. Priority can be established based on the project timeline and CWG members’ feedback.* | *Prior to each meeting, populate this column with prompt questions designed to explore the information in the ‘what’ summary. Tailor these questions to the specific issues arising. These questions should be chosen to help progress discussion towards a decision in the ‘now what’ column. Examples include: ‘do we need to consider changing this?’ ‘What further information do we need before we can make a decision?’ ‘What impact would a change have on the intervention and/or research process as a whole?’ During the meeting, additional questions and discussion points are likely to arise organically and these should be added to this column afterwards to keep a record of the decision making process.* | *Populate this column after each meeting with a list of the agreed actions, deadlines and person(s) responsible. It is important to note that actions will not necessarily be changes to the intervention. Actions, for example, may be a decision to seek more information for the next meeting (*e.g. *by adding questions to interview topic guides or asking facilitators to reflect on a specific area of delivery in supervision), or a decision to wait until the next face to face meeting before agreeing any course of action.* |
