# Supplementary material for: The collaborative working group method for pre-trial knowledge mobilisation: a qualitative evaluation of a structured process for iteratively refining a complex intervention (DAFNEplus)
Source: Pilot Feasibility Stud. 2024 Dec 21;10:154. doi: 10.1186/s40814-024-01576-3 (PMC11662412; doi:10.1186/s40814-024-01576-3)
Supplement: Supplementary file 2 — Additional file 2. Topic guide. [file 40814_2024_1576_MOESM2_ESM.docx]

**Supplementary File 2: Focus group topic guides (also used to inform individual interviews)**

**FOCUS GROUP TOPIC GUIDE 1 (between waves 1 and 2)**

What are your views on the frequency and timing of the CWG meetings?

What are your views on the format of the meetings? E.g. teleconference, face to face

How would you describe the relationship between CWG members? Has this changed over time? Why/why not?

What were the barriers to taking part?

What made it easier to take part?

What impact has the CWG had on intervention refinement? Have there been any turning points? What were these? How did the CWG support/hinder intervention refinement?

What aspects of the CWG process have been going well?

What aspects of the CWG process have not been going so well?

How could the collaborative working group process be improved?

**FOCUS GROUP TOPIC GUIDE 2 (between wave 2 and the trial)**

What are your views now about the frequency and timing of the CWG meetings? Have these changed since last time? Why/why not?

What are your views now about the format of the meetings? E.g. teleconference, face to face. Have these changed since last time? Why/why not?

How do you think the relationships between CWG members have changed over time?

Are the barriers to taking part still the same? Have these changed? If so, why?

What has made it easier to take part? Has this changed over time?

What impact has the CWG had on intervention design?

What aspects of the CWG process have been going well?

What aspects of the CWG process have not been going so well?

How could the collaborative working group process be improved?

Would you recommend the CWG process to others? Would you change anything about it?

What advice would you give to another programme grant team who were about to embark on a similar process?
